# Critical and Independent Role for SOCS3 in Either Myeloid or T Cells in Resistance to *Mycobacterium tuberculosis*

**DOI:** 10.1371/journal.ppat.1003442

**Published:** 2013-07-04

**Authors:** Berit Carow, Ann-Kathrin Reuschl, Dolores Gavier-Widén, Brendan J. Jenkins, Matthias Ernst, Akihiko Yoshimura, Benedict J. Chambers, Martin E. Rottenberg

**Affiliations:** 1 Department of Microbiology, Tumor and Cell Biology, Karolinska Institutet, Stockholm, Sweden; 2 Department of Biomedical Sciences and Veterinary Public Health, Swedish University of Agricultural Sciences and National Veterinary Institute, Uppsala, Sweden; 3 Centre for Innate Immunity and Infectious Diseases, Monash Institute of Medical Research, Monash University, Melbourne, Victoria, Australia; 4 Cell Signaling and Cell Death Division, Walter and Eliza Hall Institute of Medical Research, Melbourne, Victoria, Australia; 5 Department of Microbiology and Immunology, Keio University School of Medicine, Tokyo, Japan; 6 Center of Infectious Medicine, Karolinska Institutet, Stockholm, Sweden; Portland VA Medical Center/Oregon Health and Science University, United States of America

## Abstract

Suppressor of cytokine signalling 3 (SOCS3) negatively regulates STAT3 activation in response to several cytokines such as those in the gp130-containing IL-6 receptor family. Thus, SOCS3 may play a major role in immune responses to pathogens. In the present study, the role of SOCS3 in *M. tuberculosis* infection was examined. All *Socs3^fl/fl^ LysM cre, Socs3^fl/fl^ lck cre* (with SOCS3-deficient myeloid and lymphoid cells, respectively) and *gp130^F/F^ mice*, with a mutation in gp130 that impedes binding to SOCS3, showed increased susceptibility to infection with *M. tuberculosis*. SOCS3 binding to gp130 in myeloid cells conveyed resistance to *M. tuberculosis* infection via the regulation of IL-6/STAT3 signalling. SOCS3 was redundant for mycobacterial control by macrophages *in vitro*. Instead, SOCS3 expression in infected macrophages and DCs prevented the IL-6-mediated inhibition of TNF and IL-12 secretion and contributed to a timely CD4+ cell-dependent IFN-γ expression *in vivo*. In T cells, SOCS3 expression was essential for a gp130-independent control of infection with *M. tuberculosis*, but was neither required for the control of infection with attenuated *M. bovis* BCG nor for *M. tuberculosis* in BCG-vaccinated mice. *Socs3^fl/fl^ lck cre* mice showed an increased frequency of γδ+ T cells in different organs and an enhanced secretion of IL-17 by γδ+ T cells in response to infection. *Socs3^fl/fl^ lck cre* γδ+ T cells impaired the control of infection with *M. tuberculosis*. Thus, SOCS3 expression in either lymphoid or myeloid cells is essential for resistance against *M. tuberculosis* via discrete mechanisms.

## Author Summary

Tuberculosis is a severe disease caused by infection with the intracellular bacteria *Mycobacterium tuberculosis*. The protein “suppressor of cytokine signalling 3” (SOCS3) inhibits the responses of cells to several cytokines and growth factors that signal via the STAT3 transcription factor. Since STAT3 is a major controller of immune and inflammatory responses, we studied the role of SOCS3 in the control of infection with *M. tuberculosis*. Mice deficient in the expression of SOCS3 either in myeloid or lymphoid cells were extremely susceptible to infection with *M. tuberculosis* as measured by elevated bacterial levels, worsened pathology and reduced survival. In myeloid cells, SOCS3 hindered a detrimental role of IL-6. In absence of SOCS3, IL-6 hampered the release of IL-12 by antigen-presenting cells. In T cells, SOCS3-mediated protection was independent of IL-6 signals, and of adequate IFN-γ secretion by antigen-specific T cells. Instead, SOCS3 inhibited the *in vivo* accumulation of, and the IL-17 secretion by γδ+ T cells. γδ+ T cells accounted in part for the susceptibility to *M. tuberculosis* infection of mice with SOCS3-deficient T cells. Thus, SOCS3 controls diverse immune mechanisms of myeloid and lymphoid cells that are required for containment of *M. tuberculosis*.

## Introduction

Tuberculosis (TB), an infectious disease caused by *Mycobacterium tuberculosis*, remains a leading public health problem worldwide. The global incidence of TB is rising with 8.8 million new cases and 2 million deaths each year [1]. However, while immune responses to TB clearly show their importance in host defence, it is clear that there are still gaps in our knowledge of the host factors determining the outcome of infection.

Host responses of mycobacterial infections are primarily Th1 immune responses involving cellular effector mechanisms such as macrophage activation. IFN-γ is known to be an important mediator of mycobacterial control during clinical and experimental infections [2]. IL-12 is crucial for optimal differentiation and maintenance of IFN-γ-secreting antigen-specific Th1 cells [3], [4], and in controlling mycobacterial infections in mice and man [5], [6].

The “suppressor of cytokine signalling” (SOCS) proteins are a family of eight members that inhibit STAT activation by different receptors. SOCS proteins bind either the Janus-activated kinases (JAKs) directly inhibiting their kinase activity, or the intracellular domain of cytokine receptors thereby targeting the receptor complex for ubiquitination and subsequent proteasome-mediated degradation [7]. SOCS3 inhibits STAT3-mediated signalling by binding to the IL-6 receptor family subunit gp130, G-CSF, leptin and the IL-12 receptor [8]. Since SOCS3-deficient mice die during embryogenesis [9], [10], the role of SOCS3 *in vivo* has been studied using conditional knockdown mice. Conditional knockdown of SOCS3 in macrophages protects mice from LPS shock by reducing the secretion of IL-12 and TNF due to the enhanced anti-inflammatory effect of STAT3 [11]. However, mice with SOCS3-deficient macrophages and neutrophils succumb to toxoplasmosis, probably due to reduced IL-12 and IFN-γ responses [12]. Furthermore, SOCS3 can also inhibit STAT1 activation thereby preventing IFN-γ-like responses in cells stimulated with IL-6 [13], [14]. SOCS3 also may have several roles in T cell function. SOCS3 expression in T cells can both obstruct the differentiation of inflammatory IL-17-producing Th17 cells [15], [16] and inhibit the secretion of anti-inflammatory IL-10 and TGF-β by T cells [17] and mice with SOCS3-deficient T cells are more susceptible to infection with *Leishmania major* [17]. On the other hand, SOCS3 has also been shown to impair T-cell memory development, T cell-mediated IFN-γ secretion and LCMV virus clearance in mice [18].

In the present study, the role of SOCS3 in the outcome of infection with *M. tuberculosis* was investigated. We report that the expression of SOCS3, in either myeloid or T cells, is independently required for the control of *M. tuberculosis* infection in mice. SOCS3 expression in myeloid cells allows a proper IL-12 secretion by hampering an IL-6-mediated inhibition of IL-12 expression. SOCS3 expression in T cells reduces the frequency of γδ+ T cells in different organs and the secretion of IL-17 by + T cells in response to infection in a gp130-independent manner.

## Results

### *Socs3^fl/fl^ LysM cre* mice are highly susceptible to infection with *M. tuberculosis*

**Figure 1:**
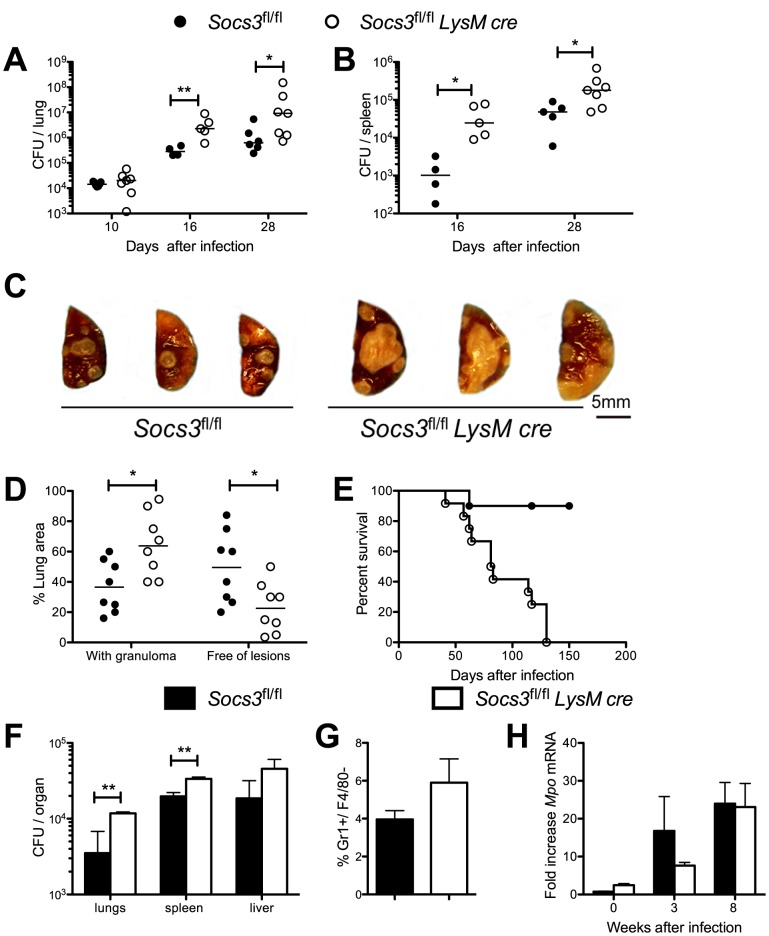
*Socs3fl/fl LysM cre* mice show higher susceptibility to infection with *M. tuberculosis*. *Socs3^fl/fl^ LysM cre* and *Socs3^fl/fl^* littermate controls were sacrificed at indicated time points after aerosol infection with M. tuberculosis and colony forming units (CFU) per lung (A) and spleen (B) were assessed. The CFU per lung of individual mice and the median per group (n≥4) at the indicated time points after infection are depicted. Differences in CFU are significant (*p<0.05 and **p<0.01 Mann Whitney U test).Gross-pathology photograph of the lungs from Socs3^fl/fl^ and Socs3^fl/fl^ LysM cre mice 8 weeks after infection with M. tuberculosis (C). Histopathological scoring of hematoxylin-eosin stained paraffin lung sections from *Socs3^fl/fl^ LysM cre* and *Socs3^fl/fl^* mice measured 4 weeks after infection with *M. tuberculosis* (D). The mean % lung area with granulomas or free of lesions ± SEM is displayed. Differences with controls are significant (n = 8 per group, *p<0.05 Student t test). The cumulative mortality of *Socs3^fl/fl^* and *Socs3^fl/fl^ LysM cre* mice (n = 10) after aerosol infection with *M. tuberculosis* is depicted (E). Survival curves are different (Log-rank test p<0.005). CFU per lung, spleen and liver in *Socs3^fl/fl^* and *Socs3^fl/fl^ LysM cre* mice (n≥5 per group) were assessed 6 weeks after infection with 10^6^ BCG i.v. (F). The median CFU and interquartile range per group are depicted. Differences in CFU are significant (**p<0.01 Mann Whitney U test). The mean percentage of Gr1+F4/80- neutrophils in the lung of *Socs3^fl/fl^* and *Socs3^fl/fl^ LysM cre* mice (n = 5 per group) 3 weeks after infection with *M. tuberculosis* ± SEM was determined by FACS analysis (G). The accumulation of myeloid peroxidase (Mpo) transcripts in lungs from mice at 0, 3 or 8 weeks after *M. tuberculosis* infection (n≥5 per group) was determined by real time PCR. The mean fold *Mpo* mRNA increase ± SEM in is depicted (H).

First, the role of *Socs3* expression in myeloid cells in the control of infection with *M. tuberculosis* was examined by using *Socs3^fl/fl^ LysM cre* mice [19]. Lungs and spleens from *Socs3^fl/fl^ LysM cre* mice showed significantly higher *M. tuberculosis* levels than *Socs3^fl/fl^* littermates at 16 and 28 days of infection (Figure 1A, B). A larger area of the lung parenchyma of *Socs3^fl/fl^ LysM cre* mice was occupied by granulomas as compared to controls 4 weeks after infection (Figure 1C, D). Furthermore, *M. tuberculosis*-infected *Socs3^fl/fl^ LysM cre* mice also showed a higher cumulative mortality (Figure 1E). *Socs3^fl/fl^ LysM cre* mice infected with the attenuated *M. bovis* BCG displayed higher bacterial levels in the lungs and spleen (but not the liver), although the differences in BCG levels with infected *Socs3^fl/fl^* littermates were not as striking as those observed after infection with *M. tuberculosis* (Figure 1F).

Since the *LysM* promoter is active in neutrophils and SOCS3 has been shown to be a negative regulator of granulopoiesis [20], [21], we studied whether the increased susceptibility to *M. tuberculosis* of *Socs3^fl/fl^ LysM cre* mice was associated to increased numbers of neutrophils at the site of infection. Comparable numbers of Gr1+/F4/80- neutrophils and similar mRNA levels of the neutrophil enzyme myeloperoxidase were detected in lungs from *M. tuberculosis*-infected *Socs3^fl/fl^ LysM cre* and control mice (Figure 1G, H).

### SOCS3-deficient macrophages are not impaired in bacterial control and can respond to IFN-γ

**Figure 2:**
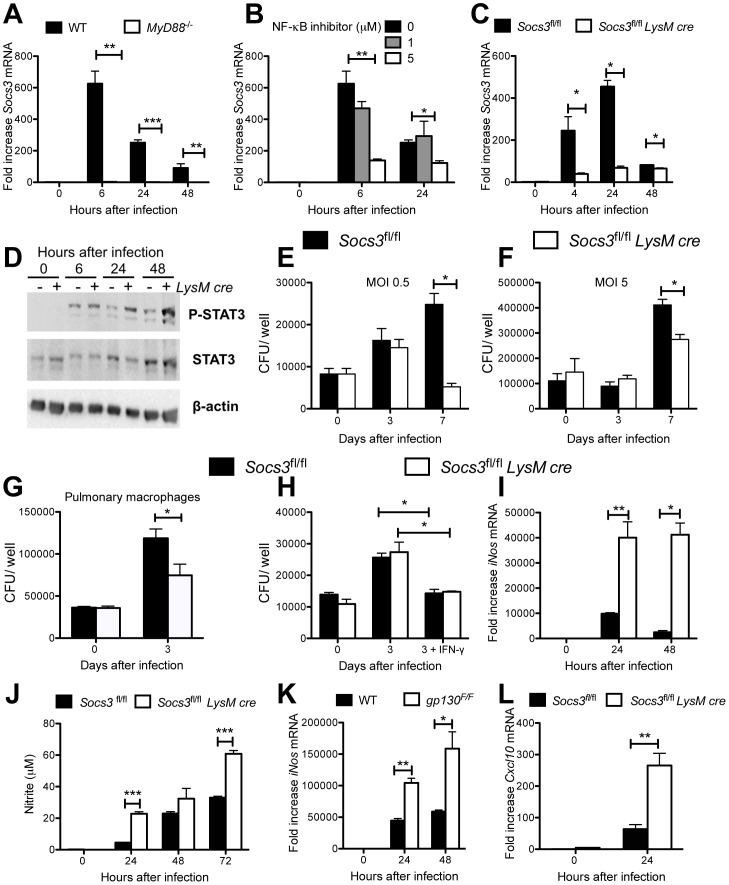
SOCS3-deficient macrophages do not display increased *M. tuberculosis* growth. Mouse BMM were infected with *M. tuberculosis* at a MOI of 5∶1 (A–C). BMM were treated with the indicated concentrations of BAY-117082 1 h before infection (B). Total RNA was isolated from *MyD88^−/−^* (A) WT (C57Bl/6) (A, B), *Socs3^fl/fl^* (C) or *Socs3^fl/fl^ LysM cre* (C) BMM at the indicated time points after infection. The mean fold *Socs3* mRNA induction ± SEM measured by real time PCR is depicted. A representative of 3 experiments is shown (C). Differences with WT (A, C) or untreated (B) BMM are significant (*p<0.05, **p<0.01, ***p<0.001 Student t test). Phosphorylated STAT3, total STAT3 and actin in lysates *Socs3^fl/fl^ LysM cre* and *Socs3^fl/fl^* BMM after infection with *M. tuberculosis* was detected by Western Blot (D). Bacterial levels were determined in *Socs3^fl/fl^ LysM cre* and *Socs3^fl/fl^* BMM after infection *M. tuberculosis* H37Rv at a MOI of 0.5∶1 (E) or 5∶1 (F). The mean CFU ± SEM from triplicate cell cultures is shown. Two independent experiments for each panel were performed. (*p<0.05 Student t test) *Socs3^fl/fl^ LysM cre* and *Socs3^fl/fl^* pulmonary macrophages were infected with *M. tuberculosis* at a MOI of 1. The CFU were determined at the indicated time points in triplicate cell cultures (*p<0.05 Student t test)(G). *Socs3^fl/fl^ LysM cre* and *Socs3^fl/fl^* BMM were infected with *M. tuberculosis* at a MOI of 5. One hundred U/ml recombinant IFN-γ were added 24 h after infection. The CFU were determined in triplicate cell cultures (H). One out of two independent experiments is shown. Differences with *Socs3^fl/fl^* BMM are significant (*p<0.05 Student t test). Total RNA was extracted from *Socs3^fl/fl^ LysM cre*, *Socs3^fl/fl^* (I, L), WT and gp130^F/F^ (K) BMM at the indicated times after infection with *M. tuberculosis* at a MOI of 5. The relative accumulation of *iNos*, *Cxcl10* and *Hprt* mRNA was measured by real time PCR. The mean fold increase of *iNos* (I, K), or *Cxcl10* (L) mRNA ± SEM in triplicate samples for each genotype and time point in one out of two independent experiments is depicted. Differences with control BMM are significant (*p<0.05 and **p<0.01Student t test). Nitrite concentrations in supernatants of *Socs3^fl/fl^ LysM cre* and *Socs3^fl/fl^* BMM at different times after infection with *M. tuberculosis*. The mean NO_2_^−^ concentration ± SEM in triplicate cultures per condition from one of two independent experiments is depicted (***p<0.001 Student t test)(J).

Next, we studied the expression and role of SOCS3 in mycobacteria-infected macrophages. Bone marrow-derived macrophages (BMM) from wild type (WT) mice showed increased accumulation of *Socs3* mRNA after infection with either *M. tuberculosis* or BCG (Figure 2A–C, S1A, B). Recognition by innate immune receptors was required for SOCS3 expression, since *Socs3* mRNA levels after infection were reduced in the Toll-like receptor adaptor molecule *MyD88* ^−/−^ BMM and BMM incubated with a NF-κB inhibitor but not in *Irf3* ^−/−^ BMM (Figure 2A, B and S1A, B). IRF3 has been shown to detrimentally affect *M. tuberculosis* infection [22]. As expected, *Socs3* mRNA levels were reduced in *M. tuberculosis*-infected *Socs3^fl/fl^ LysM cre* BMM when compared to controls (Figure 2C), and *in vitro* infection of BMM with *M. tuberculosis* stimulated STAT3 phosphorylation that was prolonged in *Socs3^fl/fl^ LysM cre* BMM (Figure 2D).

Whether a defect of macrophages to control intracellular mycobacterial growth could account for the enhanced susceptibility of *Socs3^fl/fl^ LysM cre* mice to mycobacteria was then studied. *Socs3^fl/fl^ LysM cre* BMM, pulmonary and peritoneal macrophages showed diminished intracellular levels of *M. tuberculosis* (Figure 2E–G and data not shown). The IFN-γ-mediated control of mycobacteria by macrophages is essential for the intracellular control of *M. tuberculosis*. Incubation of BMM with IFN-γ decreased the number of intracellular *M. tuberculosis*. Similar bacterial levels were measured in *Socs3^fl/fl^ LysM cre* and control BMM after incubation with IFN-γ (Figure 2H). Macrophages have been shown to kill mycobacteria through the generation of nitric oxide (NO) by the IFN-γ-regulated inducible NO synthase (iNOS) [23]. *M. tuberculosis*-infected *Socs3^fl/fl^ LysM cre* BMM contained higher *iNos* mRNA and nitrite levels than *Socs3^fl/fl^* BMM (Figure 2I, J). Similarly, infection of *Socs3^fl/fl^ LysM cre* BMM with BCG or stimulation with Pam3CSK4, an agonist for TLR2, a receptor that plays a prominent role in the initiation of responses against *M. tuberculosis* [24], led to higher NO and *iNos* mRNA levels compared to controls (Figure S1C, D).

Cells derived from *gp130^F/F^* mice, harbouring a *gp130* Y757F mutation to ablate SOCS3 binding to gp130, show an exaggerated gp130-mediated STAT3 signalling as a consequence of an impaired negative feedback loop by SOCS3 to down-modulate gp130/STAT3 signalling [25]. Similar to *Socs3^fl/fl^ LysM cre* BMM, *gp130^F/F^* BMM showed increased *iNos* mRNA and nitrite levels after infection with either *M. tuberculosis* or BCG, or stimulation with Pam3CSK4 (Figure 2K and S1E, F). Thus, the increased iNOS response of SOCS3-deficient macrophages was dependent on signalling via gp130. Similarly, the mRNA expression levels of the IFN-γ-induced chemokine CXCL10 was also increased in either *M. tuberculosis-* or BCG-infected *Socs3^fl/fl^ LysM cre* BMM (Figure 2L and S1G).

Altogether, these data demonstrated that the higher susceptibility to *M. tuberculosis* of *Socs3^fl/fl^ LysM cre* mice was not associated with a defect of BMM or pulmonary macrophages in controlling intracellular bacterial growth *in vitro*.

### SOCS3-deficient macrophages show decreased TNF and IL-12 responses during mycobacterial infection

**Figure 3:**
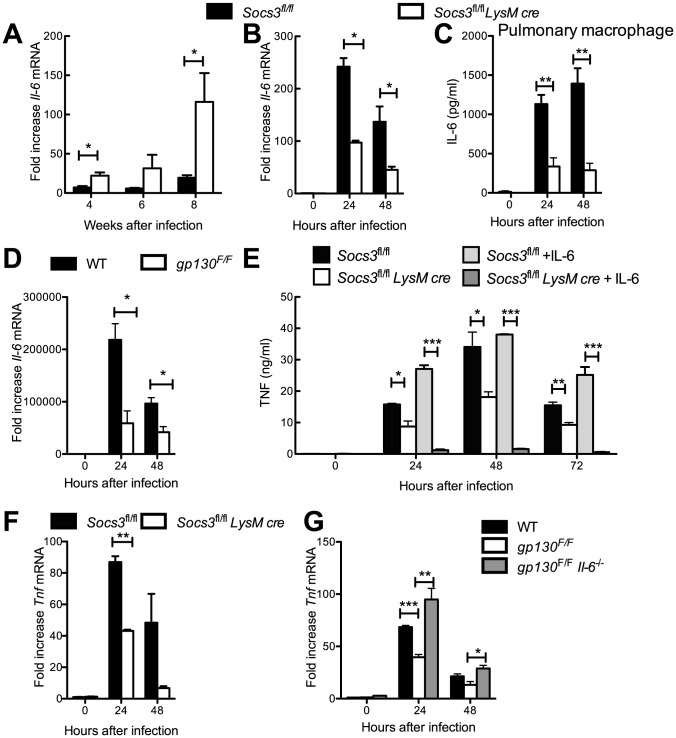
SOCS3-deficient BMM show diminished TNF secretion after infection with *M. tuberculosis*. *Socs3^fl/fl^ LysM cre* and *Socs3^fl/fl^* mice were infected with *M. tuberculosis* Harlingen via the aerosol route. Animals were sacrificed at the indicated time points after infection and the total RNA was extracted from lungs. The accumulation of *Il-6* and *Hprt* transcripts was measured by real time PCR. The mean fold *Il-6* mRNA increase ± SEM in lungs from infected mice (n≥5 per group) was calculated (*p<0.05 Student t test)(A). The levels of *Il-6* mRNA in *Socs3^fl/fl^* and *Socs3^fl/fl^ LysM cre* (B) or *gp130^F/F^* and WT (D) BMM infected with *M. tuberculosis* were determined by real time PCR. The mean fold increase of mRNA level ± SEM in triplicate independent cultures per condition compared to non-infected cultures in one out of two independent experiments is depicted (*p<0.05 Student t test). The mean IL-6 concentration ± SEM in supernatants of *M. tuberculosis*-infected *Socs3^fl/fl^* and *Socs3^fl/fl^ LysM cre* pulmonary macrophages as determined by ELISA is depicted. IL-6 secretion by BCG-infected *Socs3^fl/fl^* and *Socs3^fl/fl^ LysM cre* peritoneal macrophages is shown (C). The concentration of TNF was measured in supernatants of *M. tuberculosis*-infected *Socs3^fl/fl^* and *Socs3^fl/fl^ LysM cre* BMM co-incubated with or without 50 ng/ml of recombinant IL-6 (E). The levels of *Tnf* mRNA in *Socs3^fl/fl^* and *Socs3^fl/fl^ LysM cre* (F) or *gp130^F/F^*, *gp130^F/F^ Il-6^−/−^* and WT (G) BMM infected with *M. tuberculosis* were determined by real time PCR. The mean fold increase of mRNA level ± SEM in triplicate independent cultures per condition compared to non-infected cultures of one of two independent experiments is depicted (*p<0.05, **p<0.01, ***p<0.001 Student t test).

After infection with *M. tuberculosis*, *Il-6* mRNA levels were strikingly increased in lungs from *Socs3^fl/fl^ LysM cre* mice when compared to littermates (Figure 3A). Therefore, we evaluated whether IL-6 secretion was also elevated in mycobacteria-infected *Socs3^fl/fl^ LysM cre* BMM. Unexpectedly, we found diminished *Il-6* mRNA and protein levels in *M. tuberculosis*- or BCG-infected or in Pam3CSK4-stimulated *Socs3^fl/fl^ LysM cre* BMM, peritoneal and pulmonary macrophages as compared to controls (Figure 3B, C and S2A–C). Similarly, *gp130^F/F^* BMM expressed lower IL-6 protein and mRNA levels after infection with either *M. tuberculosis* or BCG, or stimulation with Pam3CSK4 (Figure 3D and S2D). In conclusion, although IL-6 levels are increased in lungs of infected *Socs3^fl/fl^ LysM cre* mice, SOCS3 does not impair IL-6 secretion by mycobacteria-infected BMM.

The LPS-induced production of TNF and IL-12 is reduced in SOCS3-deficient macrophages if IL-6 is added [11]. Consistent with this observation, the *Tnf* mRNA or protein accumulation was reduced in *Socs3^fl/fl^ LysM cre* BMM incubated with either *M. tuberculosis*, BCG- or Pam3CSK4 as compared to controls (Figure 3E and S2E).

Next, we studied whether IL-6 signalling accounted for SOCS3/gp130-mediated regulation of TNF levels. *Tnf* mRNA and protein levels were reduced in *M. tuberculosis-* or BCG-infected *gp130^F/F^* BMM and were partially restored in *gp130^F/F^ Il-6* ^−/−^ BMM (Figure 3G and S2F, G), indicating that SOCS3 allows proper infection-induced TNF secretion in macrophages by hampering gp130/IL-6 receptor-mediated signalling. Moreover, the co-incubation with recombinant IL-6 (rIL-6) further diminished TNF levels in *M. tuberculosis*-infected *Socs3^fl/fl^ LysM cre* but not in control BMM (Figure 3E).

**Figure 4:**
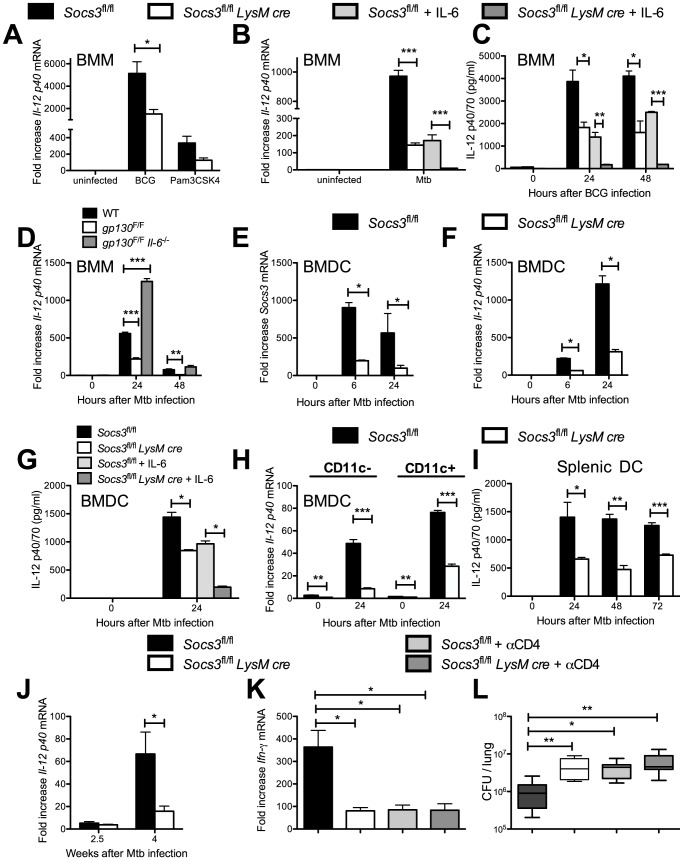
Reduced IL-12 secretion in *Socs3^fl/fl^ LysM cre* macrophages and DCs after mycobacterial infection. The levels of *Il-12 p40* mRNA were measured in triplicate cultures of *Socs3^fl/fl^ LysM cre* and *Socs3^fl/fl^* BMM infected with BCG (A) or *M. tuberculosis* (B) or treated with Pam3CSK4 for 24 h (A). Additionally, 50 ng/ml recombinant IL-6 was added to *M. tuberculosis*-infected samples (B). One out of two independent experiments is shown (*p<0.05 and ***p<0.001 Student t test). The concentration of IL-12 in supernatants from BCG-infected *Socs3^fl/fl^* and *Socs3^fl/fl^ LysM cre* BMM co-incubated with 50 ng/ml IL-6 was determined by ELISA (C). The mean IL-12 concentration ± SEM from triplicate cultures per condition in one of two independent experiments is depicted, (*p<0.05, **p<0.01, ***p<0.001 Student t test). The levels of *Il-12 p40* mRNA were measured in triplicate cultures of *gp130^F/F^*, *gp130^F/F^ Il-6^−/−^* or WT BMM infected with *M. tuberculosis* (D), (**p<0.05 and ***p<0.001 Student t test). The levels of *Socs3* (E) and *Il-12 p40* mRNA (F) in triplicate cultures of *M. tuberculosis-*infected *Socs3^fl/fl^ LysM cre* and *Socs3^fl/fl^* BMDC were determined by real time PCR. One of two independent experiments is shown, (*p<0.05 Student t test). The concentration of IL-12 was determined by ELISA in supernatants from triplicate cultures of *M. tuberculosis*-infected *Socs3^fl/fl^ LysM cre* and *Socs3^fl/fl^* BMDC co-incubated or not with 50 ng/ml IL-6 (G), (*p<0.05, Student t test). Total RNA was extracted from *M. tuberculosis*-infected CD11c+ and CD11c- *Socs3^fl/fl^* and *Socs3^fl/fl^ LysM cre* BMDC cultures 24 h after *M. tuberculosis* infection. The mean *Il-12p40* mRNA levels ± SEM levels measured by real time PCR are depicted (H). The concentration of IL-12p40 in supernatants from *M. tuberculosis*-infected *Socs3^fl/fl^* and *Socs3^fl/fl^ LysM cre* splenic DCs was determined by ELISA (I). The mean IL-12p40 ± SEM pg/ml from triplicate cultures is depicted (*p<0.05, **p<0.01, ***p<0.001 Student t test). The fold increase of *Il-12 p40* mRNA in the lungs of *M. tuberculosis*-infected *Socs3^fl/fl^ LysM cre* and *Socs3^fl/fl^* mice relative to uninfected mice is displayed (J). The data is pooled from 2 independent experiments with n≥5 animals per group in each one (*p<0.05, Student t test). Total RNA was isolated from the lungs of *Socs3^fl/fl^ LysM cre* and *Socs3^fl/fl^* mice (n≥7 per group) treated or not with CD4 cell-depleting antibodies 2.5 weeks after infection with *M. tuberculosis* (K). The mean *Ifn*-*γ* mRNA ± SEM is depicted, (*p<0.05, Student t test). Bacterial loads in lungs from *Socs3^fl/fl^* and CD4+ cell-depleted *Socs3^fl/fl^* and *Socs3^fl/fl^ LysM cre* mice (n≥5) 2.5 weeks after *M. tuberculosis infection* are shown (L). A box and whisker diagram showing the median CFU, quartiles and the 99^th^ percentiles is depicted, (*p<0.05, **p<0.01 Mann Whitney U test).

Similar to the results observed for TNF, the *Il-12 p40* mRNA and protein levels were reduced in *Socs3^fl/fl^ LysM cre* BMM after stimulation with Pam3CSK4, BCG or *M. tuberculosis* when compared to controls (Figure 4A–C). IL-12 levels were further decreased in cultures of infected mutant macrophages incubated with rIL-6 (Figure 4B, C). Accordingly, *IL-12 p40* mRNA and protein levels were reduced in *M. tuberculosis-* or BCG-infected *gp130^F/F^* BMM, and such defect was restored in infected *gp130^F/F^ Il-6^−/−^*BMM (Figure 4D and S3A).

Since IL-12 secretion by dendritic cells (DCs) is required for Th1 differentiation, we investigated whether DCs from *Socs3^fl/fl^ LysM cre* mice also showed an impaired secretion of IL-12. *Socs3* mRNA levels were reduced in *Socs3^fl/fl^ LysM cre* bone marrow-derived dendritic cells (BMDC) indicating the expression of the LysM cre recombinase (Figure 4E). The *Il-12 p40* mRNA and IL-12 protein expression by *Socs3^fl/fl^ LysM cre* BMDC after infection with *M. tuberculosis* was reduced as compared to controls (Figure 4F, G). As shown for BMM, incubation with exogenous IL-6 further diminished IL-12 secretion by *M. tuberculosis*-infected *Socs3^fl/fl^ LysM cre* BMDC (Figure 4G). In order to exclude that the diminished IL-12 secretion in BMDC cultures was due to the response of contaminant macrophages in the culture, the expression of *Il-12 p40* mRNA was tested in CD11c+ sorted cells. Both, CD11c+ and CD11c- cells showed diminished *IL-12 p40* mRNA accumulation after infection with *M. tuberculosis* compared with controls (Figure 4H). Moreover, *Socs3^fl/fl^ LysM cre* splenic DCs displayed a diminished secretion of IL-12 after *M. tuberculosis* (Figure 4I). Thus, SOCS3 expression promotes IL-12 secretion in *M. tuberculosis*-stimulated DCs.

Since IL-12 is required for IFN-γ secretion by NK cells, we tested the effect of SOCS3 expression by *M. tuberculosis*-infected DCs in the regulation of IFN-γ secretion by NK cells. Co-incubation with *M. tuberculosis*-infected splenic CD11c+ DCs induced IFN-γ secretion by NK cells. IFN-γ expression by NK cells was reduced when these cells were incubated with *Socs3^fl/fl^ LysM cre* DCs (Figure S3B, C).

Lower *Il-12 p40* mRNA levels were also found in lungs of *M. tuberculosis-*infected *Socs3^fl/fl^ LysM cre* mice compared to controls (Figure 4J) and similarly, lower *Ifn-γ* mRNA accumulation in lungs from *Socs3^fl/fl^ LysM cre* mice was detected 2.5 weeks after *M. tuberculosis* infection (Figure 4K). In order to examine whether an effect of SOCS3-deficient myeloid cells on the cytokine production by CD4+ T cells accounted for the elevated numbers of bacteria in *Socs3^fl/fl^ LysM cre* mice, we depleted CD4+ cells during *M. tuberculosis* infection by administration of anti-CD4 neutralizing antibodies (Figure S4A). CD4+ cell depletion decreased the *Ifn-γ* mRNA accumulation in lungs from *Socs3^fl/fl^* mice. The *Ifn-γ* mRNA levels in infected *Socs3^fl/fl^ LysM cre* mice were similar to those measured in CD4+ cell-depleted mice (Figure 4K). Moreover, lungs from *Socs3^fl/fl^ LysM cre* and control mice depleted of CD4+ cells showed similar bacterial levels (Figure 4L). In contrast to the decreased *Ifn-γ* mRNA expression, the frequency of CD44+ and CD62L+ CD4+ activated T cells in the lungs of *M. tuberculosis-*infected *Socs3^fl/fl^ LysM cre* and *Socs3^fl/fl^* mice was similar, and higher than in uninfected animals (Figure S4B, C). When we compared cytokine levels in *Socs3^fl/fl^ LysM cre* and *Socs3^fl/fl^* littermates at later time points after *M. tuberculosis* infection. Higher *Ifn-γ* and *iNos*, but similar *Tnf*-mRNA levels were measured in lungs from *Socs3^fl/fl^ LysM cre* compared to control mice 8 weeks after infection (Figure S4D–F).

Regulatory FoxP3+ T-cells have been shown to expand in mice with SOCS3-deficient DCs [26]. However, comparable levels of FoxP3+ CD4+ T cells were found in lungs and pulmonary lymph nodes of infected mutant and control mice (Figure S4G), suggesting that the susceptibility of *Socs3^fl/fl^ LysM cre* mice to *M. tuberculosis* is not due to higher frequencies of regulatory T cells.

Altogether, the enhanced susceptibility of *Socs3^fl/fl^ LysM cre* mice to *M. tuberculosis* could be associated to a reduced IL-12 secretion resulting in a delayed CD4+ -cell dependent IFN*-γ*-expression.

### Mice with SOCS3-deficient T cells are dramatically susceptible to infection with *M. tuberculosis*

**Figure 5:**
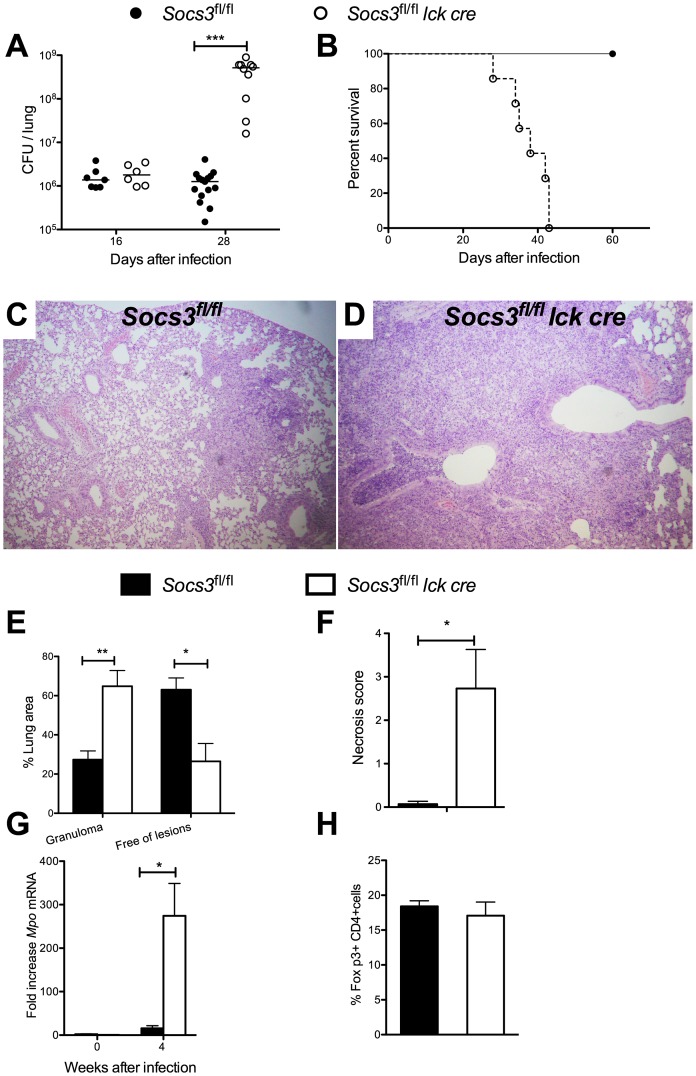
Mice with SOCS3-deficient T cells are susceptible to infection with *M. tuberculosis*. *Socs3^fl/fl^ lck cre* and *Socs3^fl/fl^* mice were sacrificed at the indicated time points after aerosol infection with M. tuberculosis, and CFU in lungs assessed (A). The CFU per lung of individual mice and the median per group (n≥5) are depicted. Results are pooled from three independent experiments. Differences in CFU are significant (***p<0.001 Mann Whitney U test). The cumulative mortality of *Socs3^fl/fl^ lck cre* and *Socs3^fl/fl^* mice (n = 10 mice per group) after aerosol infection with *M. tuberculosis* is depicted (B). Differences in survival curves are significant (Log-rank test p<0.0005) with a median survival of 38 days for *Socs3^fl/fl^ lck cre* mice. Hematoxylin-eosin stained paraffin lung sections from Socs3^fl/fl^ (C) and Socs3^fl/fl^ lck cre mice (D) and their histopathological scoring (E, F) determined 4 weeks after infection with M. tuberculosis. The mean % lung area with granulomas or free of lesions ± SEM and the mean score of lymphocytes within the granuloma or in perivascular spaces ± SEM is shown (F). Differences with controls are significant (n = 5 per group, *p<0.05 Student t test). *Socs3^fl/fl^ lck cre* and *Socs3^fl/fl^* mice were sacrificed 4 weeks after *M. tuberculosis* infection and the total RNA was extracted from lungs. The accumulation of *Mpo* and *Hprt* transcripts was measured by real time PCR (G). The mean fold increase of *Mpo* mRNA ± SEM in lungs from infected mice (n≥5 per group) was calculated. Differences with infected Socs3^fl/fl^ mice are significant (*p<0.05 Student t test). The mean frequency of FoxP3^+^ within CD4^+^ pulmonary lymph node CD3^+^ T cells from *Socs3^fl/fl^ lck cre* and *Socs3^fl/fl^* mice (n = 5 per group) was determined by FACS analysis 4 weeks after infection with *M. tuberculosis* (H).

Next, the role of SOCS3 expression by T cells in the control of infection with *M. tuberculosis* was studied. Lungs and spleens from *Socs3^fl/fl^ lck cre* mice showed higher numbers of *M. tuberculosis* bacteria 4 weeks after aerosol infection (Figure 5A and data not shown), with 500-fold higher bacterial levels in lungs compared to Socs3*^fl/fl^* littermate controls. In contrast, no differences in bacterial load were registered 2 weeks after infection (Figure 5A). Infected *Socs3^fl/fl^ lck cre* mice had a median survival of 38 days after infection while controls survived more than 200 days (Figure 5B). Four weeks after infection, *Socs3^fl/fl^ lck cre* mice displayed an increased severity of pulmonary pathology (Figure 5C–E) with granulomas containing large necrotic areas (Figure 5F) and elevated levels of neutrophil myeloperoxidase transcripts (Figure 5G). The frequency of Foxp3+ CD4+ regulatory T cells in pulmonary lymph nodes was similar (Figure 5H).

**Figure 6:**
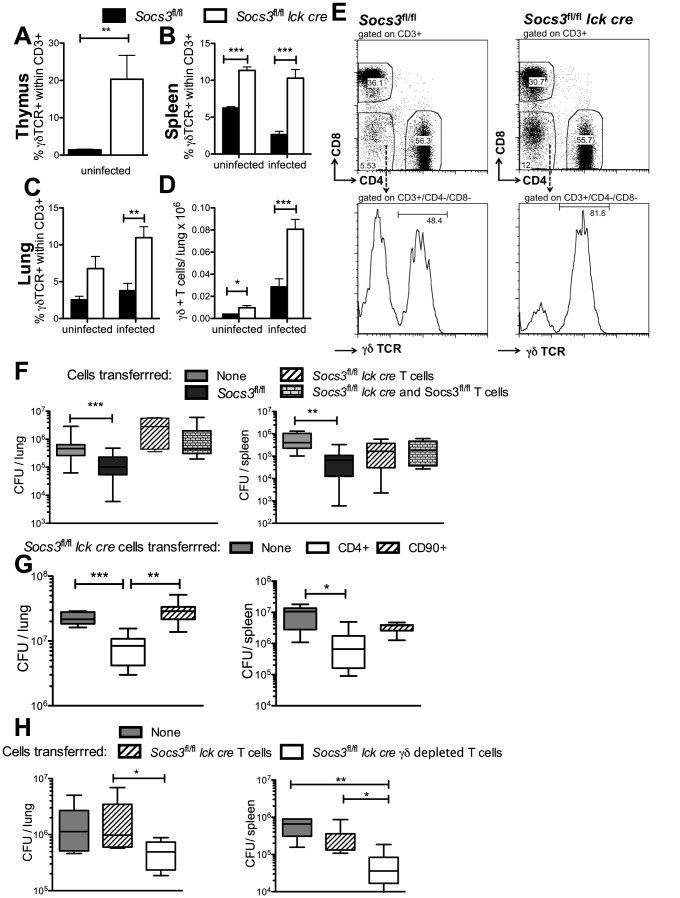
γδ+ T cell numbers are increased in organs of *Socs3^fl/fl^ lck cre* mice. The frequency of γδ+ T cells within CD3+ cells in the thymus (A), spleen (B) and lung (C) of *Socs3^fl/fl^ lck cre* and *Socs3^fl/fl^* mice obtained before or 2.5 weeks after M. tuberculosis infection were determined by FACS. Mean percentage (A–C) and the total numbers (D) of γδ+ within CD3+ T cells in the lungs ± SEM are depicted. Differences with *Socs3^fl/fl^* mice (n = 4 per group) are significant (*p<0.05, ***p<0.001 Student t test). The gating strategy and representative dot-plots for spleens of infected *Socs3^fl/fl^ lck cre* and *Socs3^fl/fl^* mice are shown (E). Two million *Socs3^fl/fl^ lck cre* or *Socs3^fl/fl^* CD90+ T cells positively selected from spleens using magnetic beads were inoculated i.v. into *Rag1^−/−^* mice. A group of animals was also inoculated with both *Socs3^fl/fl^ lck cre* and *Socs3^fl/fl^ T* cells (F). *Rag1^−/−^* mice were alternatively transferred with 1.2×10^6^ CD4+ or 2×10^6^ CD90+ *Socs3^fl/fl^ lck cre* spleen cells (G) or in a different experiment with 2×10^6^ FACS-sorted CD3+ depleted of γδ+ T cells or total CD3+ *Socs3^fl/fl^ lck cre* spleen cells (H). Two weeks after transfer, mice were infected with *M. tuberculosis* via the aerosol route. Mice (n≥6 per group) were sacrificed 4 weeks after infection. Box and whisker diagrams showing the median CFU, quartiles and the 99^th^ percentiles in lungs and spleens are depicted (F–H). Differences in CFU are significant (*p<0.05, **p<0.001, ***p<0.001 Mann Whitney U test).

Whether the susceptibility of Socs3*^fl/fl^ lck cre mice* was associated to an altered frequency of T cell populations was then evaluated. While the percentage of CD4+ and CD8+ T cells in lungs and spleens from Socs3*^fl/fl^ lck cre and Socs3^fl/fl^* mice before or after *M. tuberculosis* infection was similar (Figure S5A–D), the percentage of γδ+ T cells in the thymus, spleen and lungs of uninfected *Socs3^fl/fl^ lck cre* mice was strikingly elevated and remained high after *M. tuberculosis* infection when compared to *Socs3^fl/fl^* controls (Figure 6A–C, E). T cells accumulated in the lungs after *M. tuberculosis* infection and higher numbers of γδ+ T cells were observed in lungs from infected *Socs3^fl/fl^ lck cre* compared to *Socs3^fl/fl^* mice (Figure 6D).

The outcome of infection with *M. tuberculosis* of *Rag1* ^−/−^ mice reconstituted with control or *Socs3^fl/fl^ lck cre* T cells was then compared. Lungs and spleens from *Rag1* ^−/−^ mice transferred with Socs3*^fl/fl^* total T cells (CD90+) showed lower bacterial levels than non-transferred mice while *Socs3^fl/fl^ lck cre* T cells failed to transfer protection (Figure 6F). Moreover, the transfer of a 1∶1 mixture of *Socs3^fl/fl^ lck cre* and control T cells conferred no protection to *Rag1^−/−^* mice (Figure 6F), suggesting that SOCS3-deficient T cells can hamper *M. tuberculosis* control by wild type T cells. *Rag1^−/−^* mice transferred with CD4+ Socs3*^fl/fl^ lck cre* cells unlike those transferred with total T cells from the same mice, showed reduced *M. tuberculosis* levels compared to non-transferred controls indicating that in *Socs3^fl/fl^ lck cre* mice CD3+CD4− T cells hamper the protective ability of CD4+ cells (Figure 6G). Therefore, we examined whether γδ+ T cells could account for the suppressive activity of CD3+CD4− cells. Indeed, *Rag1^−/−^* mice transferred with γδ+ cell-depleted CD90+ *Socs3^fl/fl^ lck cre* T cells showed lower bacterial levels than those transferred with total *Socs3^fl/fl^ lck cre* T cells (Figure 6 H).

**Figure 7:**
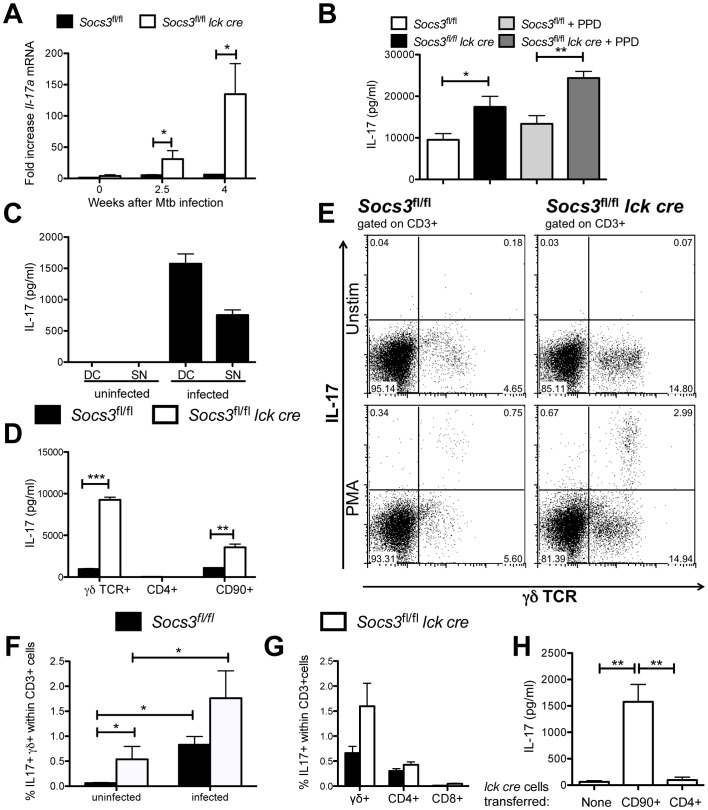
SOCS3-deficient γδ+ T cells secrete IL-17 during *M. tuberculosis* infection. *Socs3^fl/fl^ lck cre* and *Socs3^fl/fl^* mice were sacrificed before and at 2.5 and 4 weeks after M. tuberculosis infection and the total RNA was extracted from lungs. The accumulation of *Il-17a* and *Hprt* transcripts was measured by real time PCR (A). The mean fold increase of *IL-17a* mRNA ± SEM in lungs from infected mice (n≥5 per group) was calculated. One out of two independent experiments is depicted. Differences with infected Socs3^fl/fl^ mice are significant (*p<0.05 Student t test). *Socs3^fl/fl^ lck cre* and *Socs3^fl/fl^* mice were sacrificed 2.5 weeks after aerosol infection with *M. tuberculosis*. Lung cell suspensions were stimulated or not with 20 µg/ml PPD for 48 h. The IL-17 level in supernatants was determined by a cytokine bead assay (CBA) (B). The mean IL-17 concentration ± SEM (n≥6 animals per group) is depicted. Differences in cytokine concentrations are significant (*p<0.05, **p<0.01 ANOVA with Bonferroni correction). CD90+ naïve spleen T cells were co-cultured either with uninfected, BCG-infected BMDCs (DC) or with their 48 h supernatants (SN). After 72 h, the IL-17 levels in culture supernatants were measured by ELISA. A representative out of three independent experiments is shown (C). 10^5^ γδ+, CD4+ or CD90+ FACS sorted T cells from *Socs3^fl/fl^ lck cre* and *Socs3^fl/fl^* mice were co-cultured with supernatants from BCG-infected BMDCs for 72 h. The mean IL-17 concentration in supernatants from triplicate cultures ± SEM is depicted (D). Differences in cytokine concentrations are significant (**p<0.01, ***p<0.001 Student t test). The presence of IL-17-secreting cells in PMA/ionomycin-stimulated lung cell suspensions from *Socs3^fl/fl^ lck cre* or *Socs3^fl/fl^* mice before or 16 days after infection with *M. tuberculosis* was measured by FACS as described in materials and methods. Representative FACS dot plots from CD3+ gated infected lung cells before or after PMA/ionomycin stimulation are shown (E). The frequency of IL-17-secreting γδ+ within CD3+ cells in uninfected or infected mice is displayed (n = 6, *p<0.05 Mann Whitney U test) (F). The mean frequency of IL-17-secreting CD4+, CD8+ and γδ+ within CD3+ cells in lungs of infected mice (5 mice per group) ± SEM is depicted (G). *Rag1* ^−/−^ mice were infected with *M. tuberculosis* 2 weeks after inoculation with either 1.2×10^6^ CD4+ or 2×106 CD90+ *Socs3^fl/fl^ lck cre* spleen cells. Mice were sacrificed 4 weeks after infection and lung cell suspensions incubated for 48 h. The mean concentration of IL-17 in supernatants ± SEM (n = 6) is depicted (H). Differences in cytokine concentrations are significant (**p<0.01 Student t test).

Since SOCS3 expression in T cells has been shown to impair IL-17 production [27], we speculated that a differential release of IL-17 could be related with the susceptibility of *Socs3^fl/fl^ lck cre* mice to *M. tuberculosis*. Lungs from *M. tuberculosis-*infected Socs3*^fl/fl^ lck cre* mice showed higher levels of *Il-17* mRNA than controls (Figure 7A). The levels of IL-17 in supernatants from lung cells of *Socs3^fl/fl^ lck cre* mice stimulated or not with mycobacterial Purified protein derivate (PPD) were higher than controls 2.5 weeks after *M. tuberculosis* infection, when no differences in bacterial load in lungs were detected (Figure 7B). Thus, the impaired control of *M. tuberculosis*-infection in *Socs3^fl/fl^ lck cre* mice was associated with increased IL-17 levels. γδ+ T cells have been shown to dominate IL-17 secretion during infection with *M. tuberculosis* [28]. In line with this observation, the incubation of naïve spleen T cells with mycobacteria-infected BMDCs or their supernatants resulted in the secretion of IL-17 (Figure 7C). Furthermore, IL-17 was secreted by γδ+ and total (CD90+) but not by CD4+ T cells after incubation supernatants from mycobacteria-infected BMDCs. The levels of IL-17 secreted by γδ+ T cells were higher than those by similar numbers of total T cells. The IL-17 content in supernatants from γδ+ and total *Socs3^fl/fl^ lck cre* T cells was higher compared to *Socs3^fl/fl^* controls (Figure 7D).

IL-17-secreting cells were enumerated by intracellular cytokine staining in PMA/ionomycin-stimulated lung cell suspensions from *Socs3^fl/fl^ lck cre* and *Socs3^fl/fl^* mice 16 days after *M. tuberculosis* infection. The majority of IL-17-secreting lung T cells in infected mice were γδ+ rather than CD4+ or CD8+ cells (Figure 7E, G). Furthermore, *M. tuberculosis* infection stimulated the IL-17 secretion capability of γδ+ T cells (Figure 7F). However, the frequency of IL-17-secreting cells among γδ+ T cells from infected *Socs3^fl/fl^ lck cre* and *Socs3^fl/fl^* was similar, suggesting that the lack of SOCS3 does not alter the differentiation of γδ+ T cells into IL-17-secreting cells.

IL-17 was measured in supernatants from lung cell suspensions from CD4+ or CD90+ Socs3*^fl/fl^ lck cre* T cell-transferred *Rag1^−/−^* mice 4 weeks after *M. tuberculosis* infection. While IL-17 levels were strikingly higher in supernatants from mice transferred with total T cells compared with non-transferred controls, the IL-17 concentration in cultures from mice inoculated with CD4+ cells was not increased (Figure 7H). Thus, the inhibition of CD4+ cell-mediated protection in *Rag1* ^−/−^ mice transferred with CD90+ cells (Figure 6G) was associated to an increased IL-17 secretion.

**Figure 8:**
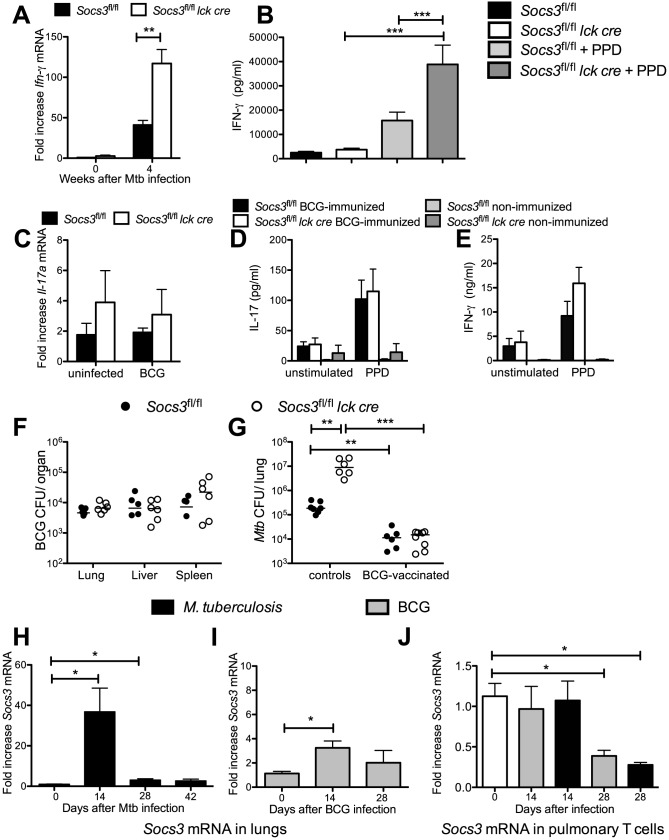
BCG-immunization protects *Socs3^fl/fl^* and *Socs3^fl/fl^ lck cre* mice equally well against *M. tuberculosis* challenge. *M. tuberculosis*-infected *Socs3^fl/fl^ lck cre* and *Socs3^fl/fl^* mice were sacrificed 4 weeks after infection and the total RNA was extracted from lungs. The accumulation of *Ifn-γ* (A) or *Hprt* transcripts was measured by real time PCR. The mean fold cytokine mRNA increase ± SEM in lungs from infected mice (n≥5 per group) was calculated. One out of two independent experiments is depicted. Differences with controls are significant (*p<0.05, **p<0.01 Student t test). *Socs3^fl/fl^ lck cre* and *Socs3^fl/fl^* mice were sacrificed 2.5 weeks after infection with *M. tuberculosis*. Lung cell suspensions were stimulated with 20 µg/ml PPD and concentrations of IFN-γ (B) in supernatants after 48 h were measured by cytokine bead assay (CBA). The mean cytokine concentration ± SEM (n≥6 animals per group) is depicted. Differences in cytokine concentrations are significant (***p<0.001 ANOVA with Bonferroni correction). Total RNA was extracted from lungs of *Socs3^fl/fl^ lck cre* and *Socs3^fl/fl^* mice 6 weeks after BCG infection. Levels of *Il-17a* (C) mRNA expression was determined by real time PCR (n = 6 mice per group). *Socs3^fl/fl^ lck cre* and *Socs3^fl/fl^* mice were immunized s.c. with 5×10^6^ heat-killed BCG and boosted after 2 weeks with 2.5×10^6^ heat-killed BCG. Mice were sacrificed four weeks after the priming, and spleen cell suspensions from immunized or non-immunized control mice were co-incubated with 15 µg/ml PPD for 72 h. The concentration of IL-17 (D) and IFN-γ (E) in the culture supernatants was determined by ELISA. The mean cytokine concentration ± SEM (n = 6 animals per group) is depicted. *Socs3^fl/fl^ lck cre* and *Socs3^fl/fl^* mice were sacrificed 6 weeks after i. v. infection with 10^6^ BCG, and the CFU per lung, spleen and liver were quantified. The individual and median CFU per group (n≥6) are depicted (F). *Socs3^fl/fl^ lck cre* and *Socs3^fl/fl^* mice were infected with 10^6^ BCG i.v. and were challenged with *M. tuberculosis* 10 weeks post BCG infection. Four weeks after *M. tuberculosis* infection, mice were sacrificed and the CFU in the lungs were quantified. The individual and median CFU per group (n≥6) are shown (G). Differences in CFU are significant (**p<0.05, ***p<0.001 Mann Whitney U test). Wild type mice were either infected with 10^6^ BCG i.v. (I, J) or via aerosol with *M. tuberculosis* Harlingen (H, J). The total RNA was isolated from lungs or pulmonary CD90+ T cells at the indicated time points. The mean fold *Socs3* mRNA increase ± SEM (n = 5 per group) was determined by real-time PCR (H–J). Differences with between groups are significant (*p<0.05 Student t test).

Lung cells from *M. tuberculosis*-infected *Socs3^fl/fl^ lck cre* mice contained higher levels of *Ifn-γ* mRNA (Figure 8A) and secreted higher levels of IFN*-*γ when stimulated with PPD than *Socs3^fl/fl^* controls (Figure 8B). Thus, the susceptibility of *Socs3^fl/fl^ lck cre* mice was not associated to an impaired secretion of IFN-γ.

The accumulation of *Il-6* mRNA was increased in lungs of *Socs3^fl/fl^ lck cre M. tuberculosis-*infected mice at 4 but not at 2.5 weeks after infection compared to controls (Figure S6A). The IL-6 levels in supernatants from *Socs3^fl/fl^ lck cre* and *Socs3^fl/fl^* lung cells obtained 2.5 weeks after infection, stimulated or not with PPD, were similar (Figure S6B). SOCS3 has been shown to regulate IL-10 secretion by T cells [17]. However, *Socs3^fl/fl^ lck cre* and Socs3*^fl/fl^* lung cells from *M. tuberculosis*-infected mice, stimulated or not with PPD, secreted similar levels of IL-10 (Figure S6C).

In contrast to results from *M. tuberculosis*-infected mice, *Il-6* and *Il-17a* mRNA levels in lungs from BCG-infected *Socs3^fl/fl^ lck cre* and *Socs3^fl/fl^* mice were similar (Figure 8C and S6D). Spleen cells from BCG-immunized *Socs3^fl/fl^ lck cre* and control mice showed comparable IL-17, IFN-γ or IL-6 secretion in response to PPD stimulation when compared with controls (Figure 8D, E and S6E). The organs from *Socs3^fl/fl^ lck cre* and Socs3*^fl/fl^* mice infected with *M. bovis* BCG contained similar bacterial levels (Figure 8F). Moreover, bacterial levels in *Socs3^fl/fl^ lck cre* and *Socs3^fl/fl^* mice challenged with *M. tuberculosis* after BCG immunization were comparable (Figure 8G).

Next, we studied whether a differing stimulation of SOCS3 expression could explain the divergent susceptibility of *Socs3^fl/fl^ lck cre* mice to *M. tuberculosis* and BCG infection. Lungs from mice infected with either *M. tuberculosis* or BCG showed higher *Socs3* mRNA levels compared to uninfected mice. *Socs3* transcript levels were higher in *M. tuberculosis*- than in BCG-infected animals (Figure 8H, I). However, *Socs3* mRNA levels in pulmonary T cells before or after infection with BCG or *M. tuberculosis* were similar (Figure 8J). Thus, different expression levels of SOCS3 in T cells do not explain the distinct susceptibility of *Socs3^fl/fl^ lck cre* mice to *M. tuberculosis* and BCG infection.

### Gp130^F/F^ mice display dramatic susceptibility to infection with *M. tuberculosis*

**Figure 9:**
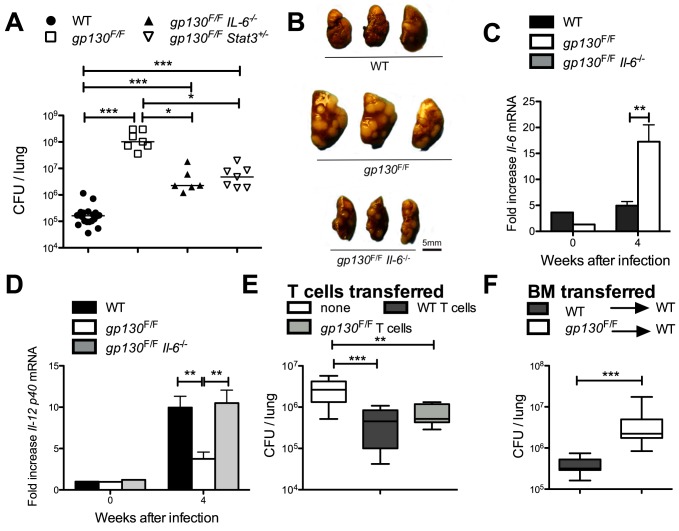
*Gp130^F/F^* mice display dramatic susceptibility to infection with *M. tuberculosis*. *Gp130^F/F^*, *gp130^F/F^ Il-6^−/−^*, *gp130^F/F^/Stat3^+/−^* and control mice were sacrificed 4 weeks after aerosol infection with *M. tuberculosis*, and CFU per lung assessed (A). The CFU in lungs of individual mice and the median per group (n≥6) are depicted. Results are pooled from two independent experiments. Differences in CFU are significant (*p<0.05, ***p<0.001 Mann Whitney U test). A gross-pathology photograph of the lungs from *gp130^F/F^*, *gp130^F/F^ Il-6^−/−^* and control mice 4 weeks after infection with *M. tuberculosis* is shown (B). *Gp130^F/F^*, *gp130^F/F^ Il-6^−/−^* and control mice were sacrificed at 4 weeks after *M. tuberculosis* infection and the total RNA was extracted from lungs. The accumulation of *Il-6* (C) and *Il-12 p40* (D) transcripts was measured by real time PCR. The mean fold cytokine mRNA increase ± SEM in lungs from infected mice (n = 5 per group) are depicted. Differences with controls are significant (**p<0.01 Student t test). 2×10^6^ CD90+ *gp130^F/F^* and control splenic T cells were inoculated i.v. into *Rag1^−/−^* mice. Two weeks after transfer, mice were infected via the aerosol route with *M. tuberculosis*. Mice were sacrificed 4 weeks after infection and the CFU in lungs determined. The median CFU (n≥10) in lungs, quartiles and the 99^th^ percentiles are depicted (E). Results are pooled from two independent experiments. Differences in CFU are significant (**p<0.01, ***p<0.001 Mann Whitney U test). The CFU in lungs of *gp130^F/F^* bone marrow→WT and WT bone marrow→ WT radiation chimeric mice were measured one month after infection with *M. tuberculosis*. A box and whisker diagram showing the median CFU (n≥8), quartiles and the 99^th^ percentiles is depicted (F). Differences in CFU are significant (***p<0.001 Mann Whitney U test).

To further characterize the function of SOCS3 in myeloid and T cells in the control of infection with *M. tuberculosis*, *gp130^F/F^* knock-in mice were used. *gp130^F/F^* mice displayed a dramatically enhanced susceptibility to *M. tuberculosis* as measured by their increased bacterial load, severity of pathology in lungs (Figure 9A, B), and increased cumulative mortality (*gp130^F/F^* mice died before 48 days after infection whereas all WT controls survived for more than 100 days). Since IL-6 mediated, at least in part, the inhibition of TNF and IL-12 secretion by SOCS3-deficient BMM and BMDC, the role of IL-6 in the susceptibility of *gp130^F/F^* mice to infection with *M. tuberculosis* was studied. We found that *gp130^F/F^Il-6* ^−/−^ as well as *gp130^F/F^Stat3^+/−^* mutant mice displayed lower levels of *M. tuberculosis* bacteria in lungs and diminished severity of pulmonary pathology when compared to *gp130^F/F^* mice, indicating that the increased susceptibility of *gp130^F/F^* mice is in part mediated by IL-6 and STAT3 activation (Figure 9A, B). Lungs from *gp130^F/F^* mice displayed higher *Il-6* and lower *Il-12 p40* mRNA accumulation than infected control mice, while *Il-12 p40* mRNA accumulation in lungs from *gp130^F/F^Il-6^−/−^* was comparable to WT mice (Figure 9C, D).

Surprisingly, lungs and spleens from *M. tuberculosis-*infected *Rag1* ^−/−^ mice transferred prior to infection with either *gp130^F/F^* or WT T cells contained similar bacterial levels, indicating that T cells play, if any, a redundant role in the gp130-mediated control of *M. tuberculosis.* Bacterial levels in mice transferred with WT or *gp130^F/F^* cells were lower than those of non-transferred controls (Figure 9E). Moreover, frequencies of γδ+ T cells in organs from WT or *gp130^F/F^* were similar (data not shown). Thus, the susceptibility of *Socs3^fl/fl^ lck cre* mice to infection with *M. tuberculosis* is mediated by receptors other than gp130.

Since T cells did not account for the increased susceptibility of *gp130^F/F^* mice and *gp130^F/F^* mice were significantly more susceptible to *M. tuberculosis* than *Socs3^fl/fl^ LysM cre* mice, we studied the relative contributions of hematopoietic and non-hematopoietic cell lineages to the susceptibility of *gp130^F/F^* mice to infection with *M. tuberculosis*. Reciprocal bone marrow (BM) radiation chimeras between WT and *gp130^F/F^* mice were generated by inoculation of BM cells into irradiated recipients. WT mice reconstituted with *gp130^F/F^* BM contained higher titers of *M. tuberculosis* in the lungs than those reconstituted with WT BM (sham chimeric mice), although differences in bacterial levels were notably lower than those observed in the *gp130^F/F^* mice (Figure 9F). Although *gp130^F/F^* recipients showed significant mortality after irradiation, the few survivors inoculated with WT BM showed very high bacterial levels, similar to those from non-irradiated *gp130^F/F^* mice (data not shown).

Thus, these data suggest that non-lymphoid, hematopoietic cells only partially account for the susceptibility of *gp130^F/F^* mice to *M. tuberculosis* and suggest a relevant role for non-hematopoietic cells in the high sensitivity of these mice to infection.

## Discussion

In the present study, we demonstrated that SOCS3 expression in lymphoid and myeloid cell populations is essential for the resistance against *M. tuberculosis* in mice via distinct mechanisms. *M. tuberculosis* and BCG infections were potent stimuli for *Socs3* expression *in vivo* and in myeloid cell populations *in vitro*.

In line with previous studies, we found that SOCS3 expression in macrophages was mediated by MyD88 and NF-κB [29]. By using *Socs3^fl/fl^ LysM cre* mice, our data suggest that myeloid SOCS3 expression contributes to a timely CD4+ cell-dependent IFN-γ-secretion rather than to improved innate effector immune mechanisms by macrophages. The observation that CD4+ cell-depleted *Socs3^fl/fl^ LysM cre* and control mice had similar bacterial loads supports this hypothesis. Moreover, SOCS3-deficient and control BMM as well as pulmonary macrophages showed comparable intracellular mycobacterial growth, and IFN-γ diminished bacterial numbers with equal efficiency in *Socs3^fl/fl^ LysM cre* and WT macrophages, as also recently demonstrated for *T. gondii* [12]. However, whether a defective IFN-γ secretion either by antigen-specific CD4+ T cells, or alternatively by NKT or CD8+ T cells underlies the susceptibility of *Socs3^fl/fl^ LysM cre* mice to infection with *M. tuberculosis* remains to be determined.

SOCS3-deficient BMM showed increased STAT3 activation and diminished secretion of TNF and IL-12 after infection with either *M. tuberculosis* or BCG. This confirms previous data showing reduced TNF and IL-12 release by *Socs3^fl/fl^ LysM cre* and *gp130^F/F^* macrophages in response to LPS, when co-incubated with IL-6 [11], [30]. Moreover, constitutively active STAT3 has been found to inhibit *Il-12 p40* mRNA accumulation in LPS-stimulated BMDC [31]. Even though the LysM promoter is primarily active in neutrophils and macrophages, LysM promoter activity in DCs has previously been shown [26]. *Socs3^fl/fl^ LysM cre* BMDC showed limited IL-12 production in response to mycobacterial stimulation and *Il-12 p40* levels were also reduced in the lungs of infected *Socs3^fl/fl^ LysM cre* and *gp130^F/F^* mice. Importantly, we found that IFN-γ levels were diminished in lungs of *Socs3^fl/fl^ LysM cre* at 16 days but not at later time points after infection with *M. tuberculosis*. This delay in the establishment of immune protective responses might underlie the increased susceptibility of *Socs3^fl/fl^ LysM cre* mice. In support of this notion, the resistance of different mouse strains to *M. tuberculosis* is associated with the timing of IFN-γ responses [32]. Although we observed a reduced secretion of IFN-γ by NK-cells during *in vitro* co-culture with *M. tuberculosis-*infected *Socs3^fl/fl^ LysM cre* and control splenic DCs, the NK cell involvement in the enhanced susceptibility of *Socs3^fl/fl^ LysM cre* mice is unlikely since NK cells were not required for controlling mycobacterial infections [33], [34].

TNF is of major importance in the control of *M. tuberculosis* [35]. Although TNF secretion by infected SOCS3-deficient macrophages is reduced, TNF expression in the lungs of *M. tuberculosis*-infected *Socs3^fl/fl^ LysM cre* mice was not diminished, suggesting that a role for TNF in the susceptibility to infection of *Socs3^fl/fl^ LysM cre* mice is unlikely.

The reduction of TNF and IL-12 levels observed in *gp130^F/F^* BMM was reversed when using *gp130^F/F^ Il-6* ^−/−^ cells, and addition of rIL-6 further diminished the release of IL-12 and TNF by either mycobacteria-infected, or Pam3CSK4-stimulated *Socs3^fl/fl^ LysM cre* BMM, suggesting that in macrophages, SOCS3 allows proper TNF and IL-12 secretion by hampering an IL-6-mediated inhibition of the secretion of these cytokines.

Lungs from *M. tuberculosis*-infected *Socs3^fl/fl^ LysM* cre and *lck cre* mice contained higher *Il-6* mRNA levels than controls. However, neither macrophages nor T cells are likely to account for the elevated IL-6 levels in *M. tuberculosis*-infected SOCS3-deficient mice. Epithelial cells, fibroblasts and adipocytes have all been shown to secrete IL-6 in response to inflammatory stimuli [36], [37]. Whether non-hematopoietic cells are major IL-6 producers during *M. tuberculosis* infection remains to be investigated.

*Socs3^fl/fl^ lck cre* mice showed a dramatically enhanced susceptibility to *M. tuberculosis* infection. However, SOCS3 expression in T cells was not required for the development of protective immune responses against *M. tuberculosis* in BCG-vaccinated mice. Thus, the requirement of SOCS3 in the control of mycobacterial infection depends on the mycobacterial species and on the immune status of the host.

Our results show a hitherto unknown role for SOCS3 controlling the frequency of γδ+ T cells in different organs before and during *M. tuberculosis* infection while frequencies of CD4+ or CD8+ T cells were not regulated by SOCS3. Moreover, γδ+ T cells impaired the transfer of protection by *Socs3^fl/fl^ lck cre* CD4+T cells, suggesting that SOCS3 inhibits a non-redundant detrimental role of γδ+ T cells in the outcome of infection with *M. tuberculosis*. The detrimental activity of SOCS3-deficient γδ+ T cells contrasts with previous reports that have shown a minor role of WT γδ+ T cells in resistance to *M. tuberculosis* [38], [39].

SOCS3 can impair the secretion of IL-17 [16], [40]. The increased IL-17 mRNA and protein levels in *Socs3^fl/fl^ lck cre* mice in *M. tuberculosis-*, but not in BCG-infected mice suggested that IL-17 levels might be causally associated to the increased susceptibility to *M. tuberculosis* infection. When γδ+ T cells but not CD4+ Socs3*^fl/fl^ lck cre* T cells were adoptively transferred in *Rag1* ^−/−^ mice, an increased IL-17 secretion by lung cells and impaired transfer of protection against *M. tuberculosis* was observed. The function of IL-17 during primary mycobacterial infections is controversial since only after high dose intratracheal infection mice deficient in IL-17 were reported to be unable to control *M. tuberculosis* infection [41]–[43]. On the other hand, IL-17 has been implicated to increase bacterial dissemination, recruitment of neutrophils and morbidity during infection with *M. tuberculosis* [44]–[46]. *Socs3^fl/fl^ lck cre* mice showed elevated levels of neutrophil-derived molecules and necrotic granulomas during *M. tuberculosis* infection. Our results confirmed a previous report indicating that γδ+T cells dominate IL-17 production during *M. tuberculosis* infection [28]. SOCS3 also hampered the secretion of IL-17 by γδ+ T cells when incubated with infected DCs or their supernatants. However, SOCS3 did not impair the development of γδ+ T cells that are capable of secreting IL-17. Thus, the increased IL-17 levels in Socs3*^fl/fl^ lck cre* mice are probably the consequence of both the increased numbers of γδ+ T cells and their unrestricted secretion of IL-17 in response to cytokines released by mycobacteria-stimulated DCs.

The increased susceptibility to *M. tuberculosis* of Socs3*^fl/fl^ lck cre* mice was not associated to an impaired IFN-γ secretion by antigen-specific T cells, suggesting that SOCS3 is not required for IFN-γ secretion by T cells and that γδ+ T cells do not modulate IFN-γ secretion by αβ+ T cells.

Since SOCS3 regulates signalling via various receptors, we investigated whether signals mediated via the gp130 receptor account for the susceptibility to *M. tuberculosis* of SOCS3 conditional knockdown animals. We found that *gp130^F/F^* mice are highly susceptible to infection with *M. tuberculosis*. The susceptibility of *gp130^F/F^* is mediated by both IL-6-dependent as well as IL-6-independent signalling events, since *gp130^F/F^Il-6* ^−/−^ mice showed lower bacterial load than *gp130^F/F^* mice but higher bacterial levels than controls. The cytokine responses of *gp130^F/F^* and *Socs3^fl/fl^ LysM cre* macrophages to mycobacterial infections were similar suggesting that the protective role of SOCS3 in myeloid cells is dependent on gp130.

IL-1β and IL-23 have been shown to stimulate IL-17 production by γδ+ T cells [47], [48], but SOCS3 impairs IL-23 signalling [27]. Thus, IL-1 β and IL-23 might mediate the elevated IL-17 secretion in *Socs3^fl/fl^ lck cre* T cells. Moreover, IL-23 signalling [49] as well as the increased frequency of γδ+ T cells in Socs3*^fl/fl^ lck cre* mice are both independent of gp130 signalling (data not shown). Accordingly, T cells from highly susceptible *gp130^F/F^* mice transferred resistance to *M. tuberculosis* as previously shown for *T. gondii* infection [50]. Since *gp130^F/F^* were more susceptible to *M. tuberculosis* than *Socs3^fl/fl^ LysM cre* mice, and lethally irradiated WT that were reconstituted with *gp130^F/F^* BM were more resistant to infection than *gp130^F/F^* or WT BM→ *gp130^F/F^* mice, we also suggest that gp130-dependent SOCS3-signalling in non-hematopoietic cells contributes to the control of infection with *M. tuberculosis*.

**Figure 10:**
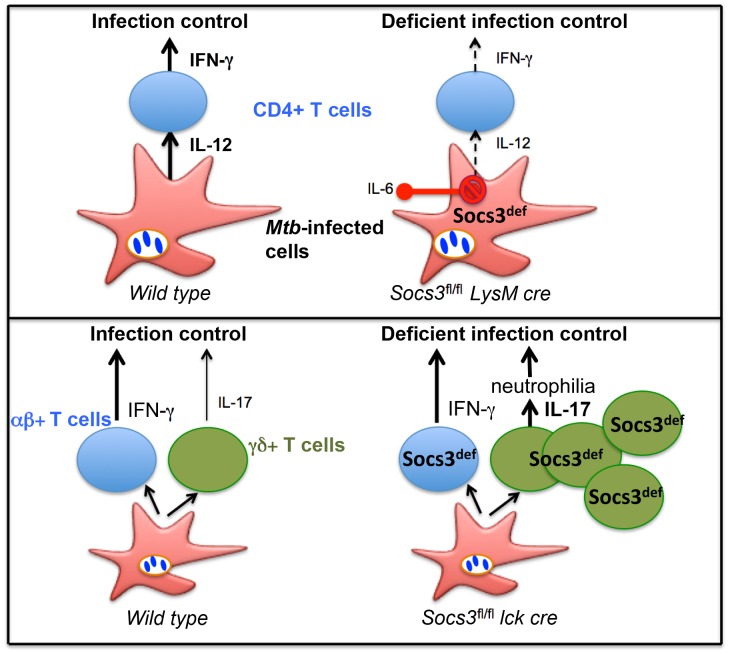
Proposed model of SOCS3-mediated roles during infection with *M. tuberculosis*. SOCS3 expression in antigen-presenting cells prevented IL-6-mediated inhibition of IL-12 secretion SOCS3 expression in T cells reduces the frequency of γδ+ T cells in different organs and the secretion of IL-17 by γδ+ T cells in response to infection in a gp130-independent manner. Expression of Socs3 in myeloid and lymphoid cell populations is critical for a proper control of *M. tuberculosis* infection.

Collectively, our data indicate that the expression of SOCS3 either in myeloid or in T cells is essential for control of *M. tuberculosis* infection (Figure 10). SOCS3 mediates protection through inhibition of IL-6/gp-130 signalling in myeloid cells, while gp130-independent, SOCS3-mediated mechanisms in T cells contribute to the control of *M. tuberculosis*.

## Material and Methods

### Ethics statement

The animals were housed and handled at the Dept. of Microbiology, Tumor and Cell Biology and the Astrid Fagreus Laboratory, Karolinska Institute, Stockholm, according to directives and guidelines of the Swedish Board of Agriculture, the Swedish Animal Protection Agency, and the Karolinska Institute (djurskyddslagen 1988:534; djurskyddsförordningen 1988:539; djurskyddsmyndigheten DFS 2004:4). The study was performed under approval of the Stockholm North Ethical Committee on Animal Experiments permit number N302/10 and N487/11. Animals were housed under specific pathogen-free conditions.

### Mice

*Socs3^fl/fl^* mice containing loxP-flanked *Socs3* alleles have been described before [51]. For a T cell-specific deletion *Socs3^fl/fl^* mice were bred with transgenic *lck cre* mice [52] and for a myeloid-specific deletion with transgenic *LysM cre* mice [19]. Offsprings were genotyped as described [51] and *Socs3^fl/fl^* littermates negative for *cre* expression were used as controls for all experiments. *Gp130^F/F^* mice possess a homozygous substitution of tyrosine (Y)_757_ to phenylalanine (F) within the common IL-6 family receptor gp130 abrogating the SOCS3 binding site. *Gp130^F/F^* mice and their corresponding compound mutant homozygous null for IL-6 (*gp130^F/F^ Il-6* ^−/−^) or heterozygous for STAT3 (*gp130^F/F^ Stat3* ^+/−^) have been described previously [53], [54], all on a mixed C57Bl/6×129/Sv background which were used as controls. *Rag1* ^−/−^ mice were generated by homologous recombination in embryonic stem cells [55] and crossed to C57Bl/6 background.

### Infection and infectivity assay

BCG Montreal and *M. tuberculosis* Harlingen and H37Rv were grown in Middlebrook 7H9 (Difco, Detroit, MI) supplemented with albumin, dextrose, catalase and, for BCG cultures, 50 µg/ml hygromycin (Sigma, St. Louis, MO). BMM and BMDC were infected at the indicated multiplicity of infection (MOI) and after 4 hours cells were washed twice with PBS to remove extracellular bacteria. Mice were infected i.v. with 1×10^6^ BCG or 250 *M. tuberculosis* Harlingen strain by aerosol using a nose-only exposure unit (In-tox Products, Uppsala, Sweden) [56]. A 15-ml suspension of 1×10^6^ *M. tuberculosis* per ml was loaded into a nebulizer, and animals inhaled the bacteria aerosol for 20 min.

Bacteria were quantified on Middlebrook 7H11 agar containing 10% enrichment of oleic acid, albumin, dextrose, catalase, 5 µg of amphotericin B per ml and 8 µg/ml polymyxin B grown for 3 weeks at 37°C.

### T cell transfer

Briefly, single-cell suspensions from spleens were selected for CD4+ or CD90+ T cells with magnetic beads (Miltenyi Biotech, Cologne, Germany) as specified by the manufacturer. When indicated, CD90+ cells were depleted of γδ+ cells by FACS sorting. 1–3×10^6^ T cells were inoculated i.v. into *Rag1* ^−/−^ mice. Two weeks after transfer, mice were infected with *M. tuberculosis* Harlingen.

### T cell depletion

Mice were injected i. p. three consecutive days with 0.5 mg/mouse of Sepharose G affinity-purified anti-CD4 (GK1.5) antibody one week before infection. CD4-specific depletion was controlled in blood using flow analysis. Two weeks after the first injection, additional 0.5 mg/mouse anti-CD4 antibody was injected to maintain CD4+ cells depleted.

### Immunization

Mice were immunized with 5×10^6^ heat-killed BCG (60 min at 80°C) s. c. and boosted after 2 weeks with 2.5×10^6^ heat-killed BCG. 4 weeks after the first injection, splenocytes from immunized and non-immunized mice were re-stimulated with 15 µg/ml PPD (Statens Seruminstitut, Copenhagen, Denmark) and supernatants collected after 72 h.

### BCG immunization and *M. tuberculosis* challenge

Mice were immunized with 1×10^6^ BCG i. v. and kept for 10 weeks before aerosol infection with *M. tuberculosis* together with non-immunized controls. Mice were sacrificed 4 weeks after *M. tuberculosis* strain Harlingen infection and bacterial loads were determined in the lungs.

### Bone marrow chimera

Recipient C57Bl/6×129/Sv and *gp130^F/F^* were irradiated 2× with 550 rad and received 5×10^6^ BM cell from either C57Bl/6×129/Sv or *gp130^F/F^* mice. Mice were kept for 3 weeks on antibiotics (Tribrissen in drinking water) and were infected with *M. tuberculosis* 8 weeks after transfer.

### Generation of mouse bone marrow-derived macrophages

Bone marrow was extracted from tibia and femurs of mice and resuspended in Dulbecco's modified Eagle's medium (DMEM) containing glucose and supplemented with 2 mM L-glutamine, 10% FCS, 10 mM Hepes, 100 µg/ml streptomycin, 100 U/ml penicillin (all from Sigma), and 30% L929 cell-conditioned medium (as a source of macrophage-colony stimulating factor). Bone marrow cells were passed through a 70 µm cell strainer, plated and incubated for 6 days at 37°C, 5% CO_2_. Bone marrow-derived macrophage (BMM) cultures were then washed vigorously to remove non-adherent cells, trypsinized, counted and cultured for one day at 37°C in 24, 12 or 6 well plates. We have previously shown by immunofluorescence staining that these BMM are F4/80+, CD14+ and Mac-3+ [57].

### Generation of mouse bone marrow-derived dendritic cells

Mouse bone marrow-derived dendritic cells (BMDC) were differentiated as previously described [58]. Briefly, bone marrow was extracted from tibia and femurs and cell suspensions cultured in RPMI-1640 medium containing 10% FCS, 100 U/ml penicillin, 100 µg/ml streptomycin and 2 ng/ml GM-CSF (Peprotech, Rocky Hill, NJ). Fresh medium and cytokine were replaced after 3 days. After six days of culture, loosely adherent cells were harvested and seeded in concentrations for infection. In some cases, harvested cells were further selected for CD11c expression with magnetic beads (Miltenyi Biotech) before seeding.

### Isolation of pulmonary macrophages

Pulmonary macrophages were isolated as previously described [59]. Briefly, lungs from *Socs3^fl/fl^* and *Socs3^fl/fl^ LysM cre* mice were dissected, digested with 1.8 U/ml dispase for 60 min at RT, followed by digestion with DNase (both form Sigma) for 30 min at 37°C. After red blood cell lysis, hematopoietic CD45+ lung cells were positively enriched using magnetic beads (Miltenyi Biotech), and pulmonary macrophages selected by plastic-adherence. Forty-eight hours after seeding to culture plates, CD45+ adherent cells were washed four times with RPMI media.

### Isolation of splenic dendritic cells

Splenic DCs were isolated as previously described [60]. Briefly, splenocyte suspensions were positively selected using anti-CD11c-coupled magnetic beads (Miltenyi Biotech). This protocol lead to a purity >95% and an approximate yield of 0.5–1×10^6^ DCs per spleen.

### In vitro stimulation of CD90+, CD4+ and γδ+ T cells

0.5×10^6^ BMDC were seeded in 500 µl medium and infected with BCG (MOI2). After 24 h, supernatants were transferred on either CD90+, CD4+ or γδ+ T cells. All cells had been separated for CD90 with magnetic beads (Miltenyi Biotech) followed by flow cytometry-based sorting (for CD4+: CD3+ and CD4+, for γδ+: CD3+, CD4−, CD8−, γδ+ TCR). 72 h after transfer, supernatants were harvested and IFN-γ and IL-17 concentrations were determined by ELISA.

### Real time PCR

Transcripts were quantified by real time PCR as previously described [56]. *Hprt* was used as a control gene to calculate the ΔC_t_ values for individual samples. The relative amount of cytokine/*Hprt* transcripts was calculated using the 2^−(ΔΔCt)^ method. These values were then used to calculate the relative expression of cytokine mRNA in uninfected and infected cells and tissues.

### Cytokine determinations

Concentrations of cytokines in supernatants of stimulated cells were determined either by using cytometric bead array (CBA) mouse Th1/Th2/Th17 cytokine kit (BD Biosciences, San Jose, CA) or by enzyme-linked immunosorbent assays (ELISA) for IL-6, IFN-γ, TNF (BD Biosciences), IL-12 and IL-10 (eBioscience, San Diego, CA) and IL-17 (R&D systems, Minneapolis, MN) following the manufacturers' recommendations.

### Flow cytometry and intracellular cytokine staining

Lungs were perfused with PBS through the heart before removal from mice. Following digestion with Collagenase D and DNase I, erythrocytes were lysed and single-cell suspensions prepared by filtering lung tissue through 70-µm nylon cell strainers. Single spleen cell suspensions were obtained by mechanical disruption, lysis of erythrocytes and straining over a 70-µm nylon mesh. Lung cells and splenocytes were stained for CD3, CD4, CD8 and γδ TCR (all eBioscience) or F4/80 and Gr1 (BD Biosciences) and fixed before acquisition.

For determination of IL-17-producing cells, lung cells were incubated with 50 ng/ml phorbol myristate acetate (PMA) and 2 µg/ml ionomycin (Sigma) in presence of brefeldin A (5 µg/ml) for 6 hours, stained with cell population-specific antibodies, fixed, permeabilized using leukocyte permeabilization reagent IntraPre (Immunotech, Marseille, France) and stained with anti-IL-17a (eBioscience).

CD11c+ splenic DCs were infected with *M. tuberculosis* H37Rv MOI5 for 4 hours, washed and cultured overnight with DX5+ NK cells separated from spleens with magnetic beads (Miltenyi Biotech). The next day, cells were treated with brefeldin A (5 µg/ml) for 4 hours, followed by a FACS stain for DX5 (PE, BD Biosciences) and intracellular IFN-γ (eBioscience).

Data were acquired in CyAn ADP flow cytometer (Beckman Coulter) and analyzed using FlowJo software (Tree star Inc., Ashland, OR).

### Western blot

*M. tuberculosis*-infected and uninfected BMM were lysed and separated on 10% separating/5% stacking SDS-polyacrylamide gels. Samples were then transferred onto nitrocellulose membranes (BioRad, Hercules, CA) by electroblotting at 100 V, 250 mA for 80 min. Immunostaining was performed using polyclonal rabbit anti-phosphorylated (Tyr701) STAT3, total STAT3 (Cell signaling technology, Beverly, MA) or anti-actin (Sigma). Membranes were then washed and incubated with horse-radish peroxidase-conjugated polyclonal goat anti-rabbit immunoglobulin (DAKO) and developed using ECL-Plus (Amersham Biosciences, Buckinghamshire, UK) and photographed using a Fuji intelligent dark box II digital camera.

### Histopathological analysis

Formalin fixed left lungs of mice experimentally inoculated with *M. tuberculosis* were blocked on paraffin. From each lung sample 4 sections were obtained, one longitudinal along the long axis of the lobe and 3 across/transversal of the remaining piece of lung.

The blocks were processed and sections were stained with haematoxylin-eosin. All sections were interpreted by the same pathologist (D. G-W.) and scored semi-quantitatively, blinded to the variables of the experiment.

The following features were scored:

- Lung area occupied with granulomas (% of the total area of the section)
- Lung area free of lesions or area of healthy lung (% of the total area of the section)
- Extension of necrosis, raging from 0: no necrosis observed to 4: extensive necrosis and necrotic centers with mineralization.

## Supporting Information

Figure S1 **Increased NO release in SOCS3-deficient BCG-infected BMM.** Mouse BMM were infected with BCG (A, B). BMM were treated with the indicated concentrations of BAY-117082 1 h before BCG infection (B). Total RNA was isolated from *Irf3^−/−^*, *MyD88^−/−^* and WT (C57Bl/6) BMM at the indicated time points after infection. A MOI of 5∶1 was used all over. Real time PCR was used to obtain duplicate determinations of *Socs3* and *Hprt* mRNA from triplicate samples for each group and time point. The mean fold *Socs3* mRNA induction ± SEM is depicted. Differences with control BMM are significant (*p<0.05, **p<0.01, **p<0.001 Student t test). Nitrite concentrations in supernatants of *Socs3^fl/fl^ LysM cre* and *Socs3^fl/fl^* (C) *gp130^F/F^* and WT (E) BMM were measured by Griess assay at the indicated time points after incubation with Pam3CSK4 or infection with BCG. The mean NO_2_^−^ concentration ± SEM in triplicate cultures per condition of one of two independent experiments is depicted (C, E). Differences with WT BMM are significant (**p<0.01 and ***p<0.001 Student t test). Total RNA was extracted from *Socs3^fl/fl^ LysM cre*, *Socs3^fl/fl^* (D, G) *gp130^F/F^* and WT (F) BMM at the indicated times after infection with BCG or stimulation with Pam3CSK4. The relative accumulation of *iNos* (D, F), *Cxcl10* (G) and *Hprt* was measured by real time PCR. The mean fold increase of cytokine mRNA ± SEM in triplicate cultures for each genotype and time point is depicted. Differences with WT BMM are significant (*p<0.05, **p<0.01 Student t test).
(EPS)

Figure S2 **Diminished IL-6 and TNF-secretion by BCG-infected SOCS3-deficient BMM.** IL-6 concentration was determined in supernatants of BCG-infected BMM (A, D) or peritoneal macrophages (B). The mean IL-6 in *Socs3^fl/fl^* and *Socs3^fl/fl^ LysM cre* (A, B) and *gp130^F/F^* and WT (D) BMM ± SEM as determined by ELISA is depicted. Differences with control BMM are significant (*p<0.05 and ***p<0.001 Student t test). The mean levels of *Il-6* mRNA in *Socs3^fl/fl^* and *Socs3^fl/fl^ LysM cre* BMM either infected with BCG or treated with Pam3CSK4 were determined by real time PCR in triplicate independent cultures per condition and compared to non infected cultures (C). One representative of two independent experiments is shown. Differences with control BMM are significant (**p<0.01Student t test). The mean levels of *Tnf* mRNA in *Socs3^fl/fl^* and *Socs3^fl/fl^ LysM cre* (E) and *gp130^F/F^*, *gp130^F/F^Il6^−/−^* and *WT* (G) BMM either infected with BCG or treated with Pam3CSK4 determined by real time PCR in triplicate independent cultures per condition compared to non infected cultures is depicted. Differences with control BMM are significant (**p<0.01Student t test). The mean TNF concentration in supernatants of BCG-infected *gp130^F/F^*, *gp130^F/F^Il6^−/−^* and *WT* BMM ± SEM as determined by ELISA in triplicate cultures per condition (F). Differences with control BMM are significant (**p<0.01Student t test).
(EPS)

Figure S3 **Diminished IL-12-secretion by BCG-infected SOCS3-deficient BMM.** The levels of *Il-12 p40* mRNA were measured in triplicate cultures of BCG-infected *gp130^F/F^*, *gp130^F/F^ Il-6^−/−^* and WT BMM (A). Differences with WT BMM are significant (*p<0.05, **p<0.01 Student t test). Isolated *Socs3^fl/fl^ LysM cre* and *Socs3^fl/fl^* CD11c+ splenic DCs were infected with *M. tuberculosis* (MOI5) and cultured overnight with NK cells. The next day, cells were treated with brefeldin A, followed by FACS staining for DX5 and intracellular IFN-γ The mean percentage of IFN-γ+ NK cells ± is depicted (B). Differences with *Socs3^fl/fl^* splenic DC are significant (n = 5, *p<0.05, Student t test). Representative FACS contour plots from co-cultures of NK cells with either infected or uninfected *Socs3^fl/fl^ LysM cre* and *Socs3^fl/fl^* splenic DCs are shown (C).
(EPS)

Figure S4 **Immune response parameters of Socs3^fl/fl^ LysM cre and Socs3^fl/fl^ mice after infection with M. tuberculosis.** *In vivo* depletion of CD4+ cells after inoculation with GK1.5 anti-mouse CD4 monoclonal antibodies. The FACS plots of CD3+ gated spleen cells from anti-CD4-treated or untreated *Socs3^fl/fl^ LysM cre* and *Socs3^fl/fl^* mice 2.5 weeks after *M. tuberculosis* infection are shown (A). These plots are representative for 5 mice analysed per group. *Socs3^fl/fl^ LysM cre* and *Socs3^fl/fl^* lung cells were stained for CD3, CD4, CD44 and CD62L. Representative FACS dot-plots of lung cells before or 2.5 weeks after *M. tuberculosis* infection in which staining for CD44 and CD62L on CD3+ CD4+ cells is shown (B). The mean frequency of CD44+/CD62L- cells within the CD3+CD4+ T cell population ± SEM (n = 5 per group) is displayed (C). *Socs3^fl/fl^ LysM cre* and *Socs3^fl/fl^* mice were infected with *M. tuberculosis* via the aerosol route. Animals were sacrificed at the indicated time points after infection and the total RNA was extracted from lungs. The accumulation of *Ifn-γ* (D), *Tnf* (E) and *iNos* (F) transcripts was measured by real time PCR. The mean fold cytokine mRNA increase ± SEM in lungs from infected mice (n =  at least 5 per group) was calculated. Differences with controls are significant (* p<0.05 Student t test). The mean frequency of FoxP3+ within CD4^+^ T cells in the pulmonary lymph nodes and lungs from mice (n = 5 mice per group) 6 weeks after infection with *M. tuberculosis* was determined by FACS (G).
(EPS)

Figure S5 **T cell subpopulations in Socs3^fl/fl^ lck cre mice.** Lung and spleen cells obtained from *Socs3^fl/fl^ lck cre* and *Socs3^fl/fl^* mice before or 2.5. weeks after infection with *M. tuberculosis* were stained for CD3, CD4 and CD8 (A–D). Mean frequencies of CD4+ (A, C) and CD8+ (B, D) CD3+ T cells in lungs (A, B) and spleens (C, D) ± SEM are shown.
(EPS)

Figure S6 **Secretion of IL-6 in Socs3^fl/fl^ lck cre mice during mycobacterial infection.** *M. tuberculosis-*infected *Socs3^fl/fl^ lck cre* and *Socs3^fl/fl^* mice were sacrificed at the indicated time points and the total RNA was extracted from lungs. The accumulation of *Il-6* (A) transcripts was measured by real time PCR. The mean fold mRNA increase ± SEM in lungs from infected mice (n≥5) was calculated. Differences with controls are significant (*p<0.05 Student t test). Lung cell suspensions from *Socs3^fl/fl^ lck cre* and *Socs3^fl/fl^* mice at 2.5 weeks after infection with *M. tuberculosis* were stimulated with 20 µg/ml PPD. The content of IL-6 (B) and IL-10 (C) in supernatants analysed by a cytokine bead array (CBA) 48 h after stimulation. The mean cytokine concentration ± SEM (n = 6 animals per group) is depicted. Levels of *Il-6* mRNA expression was determined by real time PCR in lungs of *Socs3^fl/fl^* and *Socs3^fl/fl^ lck cre* mice, before and 6 weeks after BCG infection (n = 6 per group) (D). *Socs3^fl/fl^* and *Socs3^fl/fl^ lck cre* mice were immunized s.c. with 5×10^6^ heat-killed BCG and boosted after 2 weeks with 2.5×10^6^ heat-killed BCG. After 2 weeks, mice were sacrificed and splenocytes from immunized or non-immunized control mice were co-incubated or not with 20 µg/ml PPD for 72 h. The mean IL-6 concentration ± SEM (n = 6 animals per group) in lung cell supernatants was determined by ELISA (E).
(EPS)
